# Relationship of foot anthropometry with Modified Pirani score during Ponseti correction of idiopathic congenital talipes equinovarus: a prospective observational study

**DOI:** 10.1007/s00402-026-06467-1

**Published:** 2026-08-20

**Authors:** Meera Suresh, Deepak John Victor Pinto, Pranav Rajasekharan

**Affiliations:** Department of Orthopaedics, Kasturba Medical College Mangalore, Manipal Academy of Higher Education, Manipal, India

**Keywords:** Congenital talipes equinovarus, Clubfoot, Ponseti method, Pirani score, Anthropometry, Pediatric orthopaedics, Foot measurements, Clubfoot correction

## Abstract

**Background:**

The Ponseti method is the gold standard treatment for idiopathic congenital talipes equinovarus (CTEV). Clinical monitoring during correction primarily relies on the Modified Pirani score, which evaluates deformity severity based on clinical findings. However, objective quantitative assessment of morphological foot changes during treatment remains limited. This study aimed to evaluate the relationship between serial foot anthropometric measurements and Modified Pirani scores during Ponseti correction of idiopathic clubfoot.

**Methods:**

A prospective observational cohort study was conducted in 30 children with idiopathic unilateral CTEV undergoing Ponseti correction at a tertiary care center between June 2024 and February 2026. Five anthropometric parameters were measured weekly for eight weeks: great toe to midheel distance (GT-MH), little toe to midheel distance (LT-MH), second toe to midheel distance (ST-MH), medial malleolus to midheel distance (MM-MH), and lateral malleolus to midheel distance (LM-MH). Modified Pirani scores were assessed simultaneously by two independent observers. Pearson correlation analysis and inter-observer reliability testing were performed.

**Results:**

Significant improvement was observed in all anthropometric parameters and Pirani scores over the study period (p < 0.001). Mean GT-MH increased from 80.73 ± 3.36 mm to 87.56 ± 3.41 mm, LT-MH from 65.36 ± 2.04 mm to 70.22 ± 2.05 mm, and MM-MH from 26.19 ± 0.79 mm to 30.85 ± 0.78 mm. LM-MH decreased from 28.40 ± 0.56 mm to 25.25 ± 0.51 mm, reflecting correction of hindfoot varus. Pirani score improved from 5.33 ± 0.24 to 0.03 ± 0.18. Strong inverse correlations were observed between Pirani score and GT-MH (r = -0.56), LT-MH (r = -0.61), ST-MH (r = -0.71), and MM-MH (r = -0.88), while LM-MH demonstrated a positive correlation (r = 0.71). Inter-observer reliability was excellent, with ICC values ranging from 0.9956 to 1.000. Percutaneous Achilles tenotomy was required in 80% of patients.

**Conclusions:**

Serial anthropometric assessment demonstrates significant correlation with Modified Pirani scoring during Ponseti correction of idiopathic clubfoot. Anthropometric measurements provide objective and reproducible quantitative data that complement clinical scoring and may improve monitoring of deformity correction during treatment.

**Trial registration:**

Institutional Ethics Committee approval was obtained prior to commencement of the study.

## Background

Congenital talipes equinovarus (CTEV) (clubfoot) is one of the most common congenital musculoskeletal deformities presenting to orthopedic departments in all parts of the world․ The classical deformity includes equinus, varus, adductus, cavus, Calcaneopedal Unit (CPU) rotation, and forefoot supination, resulting in a foot that is rigid, inverted, and plantarflexed [1]. The birth prevalence of clubfoot is reported to be between 0․51 and 2․03 per 1000 live births in low- and middle-income countries [2]․ Without treatment‚ lifelong disability‚ in the form of abnormal gait (walking)‚ pain‚ callosities‚ difficulty in wearing shoes and psychosocial handicap can occur [3]․

Historically, treatment of clubfoot relied heavily on extensive surgical soft-tissue release procedures. Although the deformity was corrected immediately‚ long-term follow-up of children who underwent wide-ranging surgical release consistently found stiffness‚ weakness‚ pain‚ deformity recurrence‚ and degenerative joint disease [4]․ The Ponseti method is now considered the gold standard for the treatment of idiopathic clubfoot. The technique is based on the biomechanical concept of the Calcaneopedal Unit (CPU), in which the calcaneus, navicular, cuboid, and forefoot move as a single functional unit beneath the talus. During sequential manipulation and casting, the CPU is gradually abducted and externally rotated around the head of the talus, correcting cavus, adduction, varus, and ultimately equinus while preserving foot mobility and function. This biomechanical approach has markedly reduced the need for extensive surgical release, with major surgery now required in only a small proportion of cases [5–7]․

Monitoring of correction during Ponseti treatment is primarily based on clinical scoring systems, particularly the Modified Pirani score. This score is based on six clinical features of deformity severity providing total scores between 0 (normal) and 6 (severely deformed)․ It is commonly used due to its simplicity‚ reproducibility and practicality of estimating progress of treatment and predicting need for tenotomy [8]․ However‚ the Pirani score is mostly a clinically-based estimate of foot appearance and severity of soft tissues contracture but not a measure of anatomic correction [9]․

Anthropometry may provide additional objective evidence of deformity correction during the progressive correction process of Ponseti treatment․ Great toe to midheel distance‚ little toe to midheel distance‚ and malleolar distances may reflect correction of deformity through changes in forefoot and hindfoot alignment during serial casting [10, 11]․

Though Pirani scoring‚ predictors of tenotomy‚ and predictors of Ponseti correction outcome have been studied‚ the literature on serial anthropometric measurements of clubfeet in the correction process is sparse‚ particularly in the Indian population․ Objective anthropometry may help in improving reproducibility and evaluation of the correction and may therefore be used as a supplement to clinical scoring․

The purpose of the current study was to analyze and assess the serial anthropometric changes occurring during Ponseti correction for idiopathic clubfoot and to determine whether these correlate with the Modified Pirani scores.

## Methods

### Study design and setting

This prospective observational cohort study was conducted in the Department of Orthopaedics from June 2024 to February 2026 after obtaining approval from the Institutional Ethics Committee.

### Study population

A total of 30 pediatric patients with idiopathic unilateral congenital talipes equinovarus (CTEV) undergoing Ponseti correction were included in the study.

### Inclusion and exclusion criteria

Children aged less than 2 years with idiopathic unilateral CTEV undergoing Ponseti treatment as the primary mode of management were included after obtaining written informed parental consent. Excluded were those with syndromic or neuromuscular clubfoot‚ previous clubfoot surgery‚ previous Ponseti treatment‚ systemic disease affecting bone growth‚ or incomplete follow-up․

### Sample size

Using the formula for the estimation of the correlation coefficient‚ the sample size was calculated with an expected correlation coefficient of ± 0․5‚ 95% confidence interval‚ and 80% statistical power for 30 patients․

Only unilateral idiopathic clubfoot cases were included in order to maintain a homogeneous study cohort and ensure that each participant contributed a single affected foot to the analysis. This approach avoided the statistical dependence associated with bilateral clubfoot, in which both feet from the same patient are biologically and clinically correlated, thereby preserving the assumption of independent observations and improving internal validity.

### Data collection

**Fig. 1:**
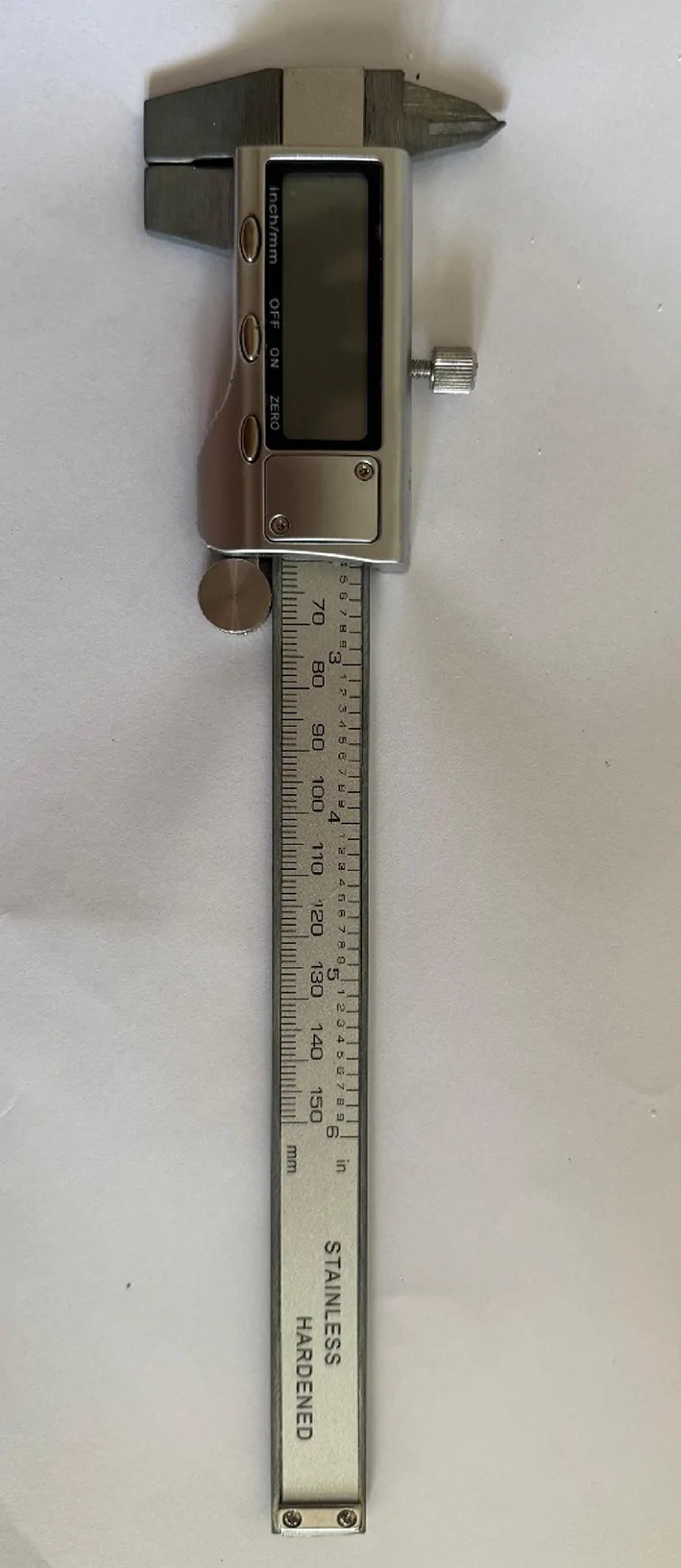
Digital Sliding Caliper

Baseline demographics and clinical characteristics (age‚ sex‚ laterality‚ birth history‚ family history) were obtained at enrollment․ Anthropometric measurements were performed using a digital sliding caliper (Fig. 1), a precision measuring instrument used to obtain accurate linear anthropometric measurements, which was modified for safe infant use by smoothing and rounding the jaw edges. The modification preserved jaw alignment and measurement accuracy. Before each assessment, the caliper was inspected and checked for zero error. Measurements were obtained with light contact over anatomical landmarks, avoiding soft tissue compression. The Modified Pirani score and anthropometric measurements were recorded at each visit as treatment outcome was noted․

### Anthropometric measurements

**Fig. 2:**
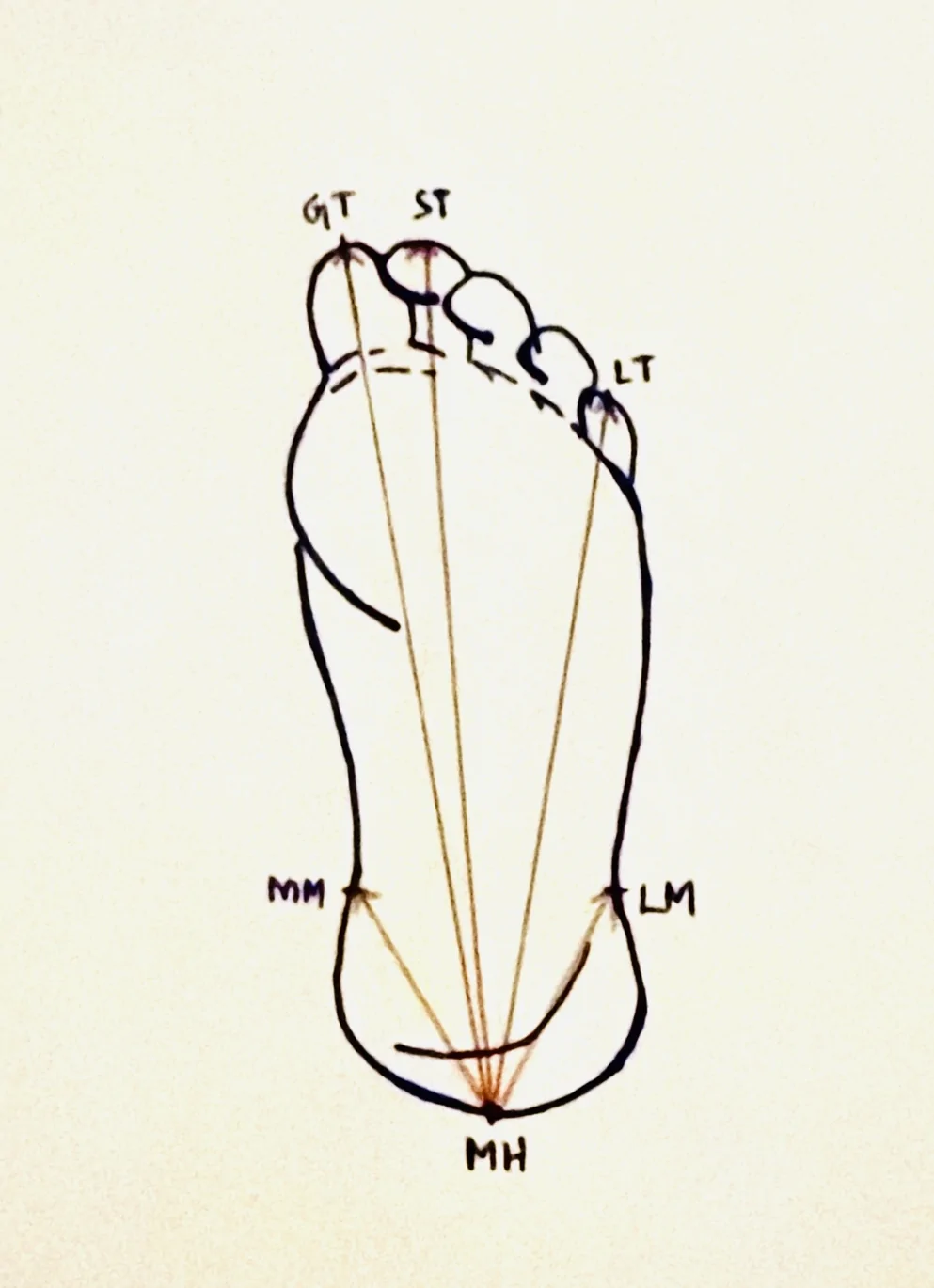
Anthropometric measurements on the foot

To ensure standardized measurements, the foot was positioned in a neutral, plantigrade position without applying excessive force. Anthropometric measurements were obtained immediately after cast removal and before manipulation and application of the subsequent cast at each weekly visit. The midheel was defined as the midpoint of the posterior aspect of the heel at the level of the calcaneal tuberosity. All measurements represented straight-line linear distances between predefined anatomical landmarks and were obtained using the same anatomical landmarks and measurement technique after cast removal at each assessment. Anthropometric evaluation included five parameters measured using digital calipers: great toe to midheel distance (GT-MH), little toe to midheel distance (LT-MH), second toe to midheel distance (ST-MH), medial malleolus to midheel distance (MM-MH), and lateral malleolus to midheel distance (LM-MH) (Fig. 2). Measurements were performed independently by two blinded investigators using the same digital calipers throughout the study to minimize inter-observer variability, and the mean values were used for statistical analysis.

ST-MH was used as an internal reference because it is relatively less affected by cavus and medial column shortening, thereby providing an estimate of physiological foot growth during treatment.

### Modified Pirani score

Severity of the deformity was assessed using the Modified Pirani scoring system, in which six clinical parameters were scored from 0 to 1, producing a total score ranging from 0 to 6, with higher scores indicating greater deformity severity.

Percutaneous Achilles tenotomy was performed following the final corrective cast, when residual equinus persisted despite correction of the other deformity components, in accordance with the standard Ponseti protocol.

### Statistical analysis

Statistical analysis was performed using SPSS version 22․0 (IBM Corp․‚ Armonk‚ NY‚ USA)․ Continuous variables are expressed as mean ± standard deviation․ Categorical variables are presented as frequencies and percentages․ Intergroup comparisons of serial anthropometric measurements and Pirani scores were performed using paired t-test and repeated measures ANOVA․ Anthropometric parameters were correlated with Modified Pirani scores using Pearson correlation․ Inter-observer reliability was assessed using correlation coefficients. A* p*-value < 0.05 was considered statistically significant.

## Results

### Demographic characteristics

Among the 30 patients included in the study, 16 (53.3%) were male and 14 (46.7%) were female. Left-sided involvement was observed in 17 patients (56.7%), while 13 patients (43.3%) had right-sided involvement.

### Anthropometric progression during treatment

Significant improvement was observed in all anthropometric parameters between Week 1 and Week 8.

**Fig. 3:**
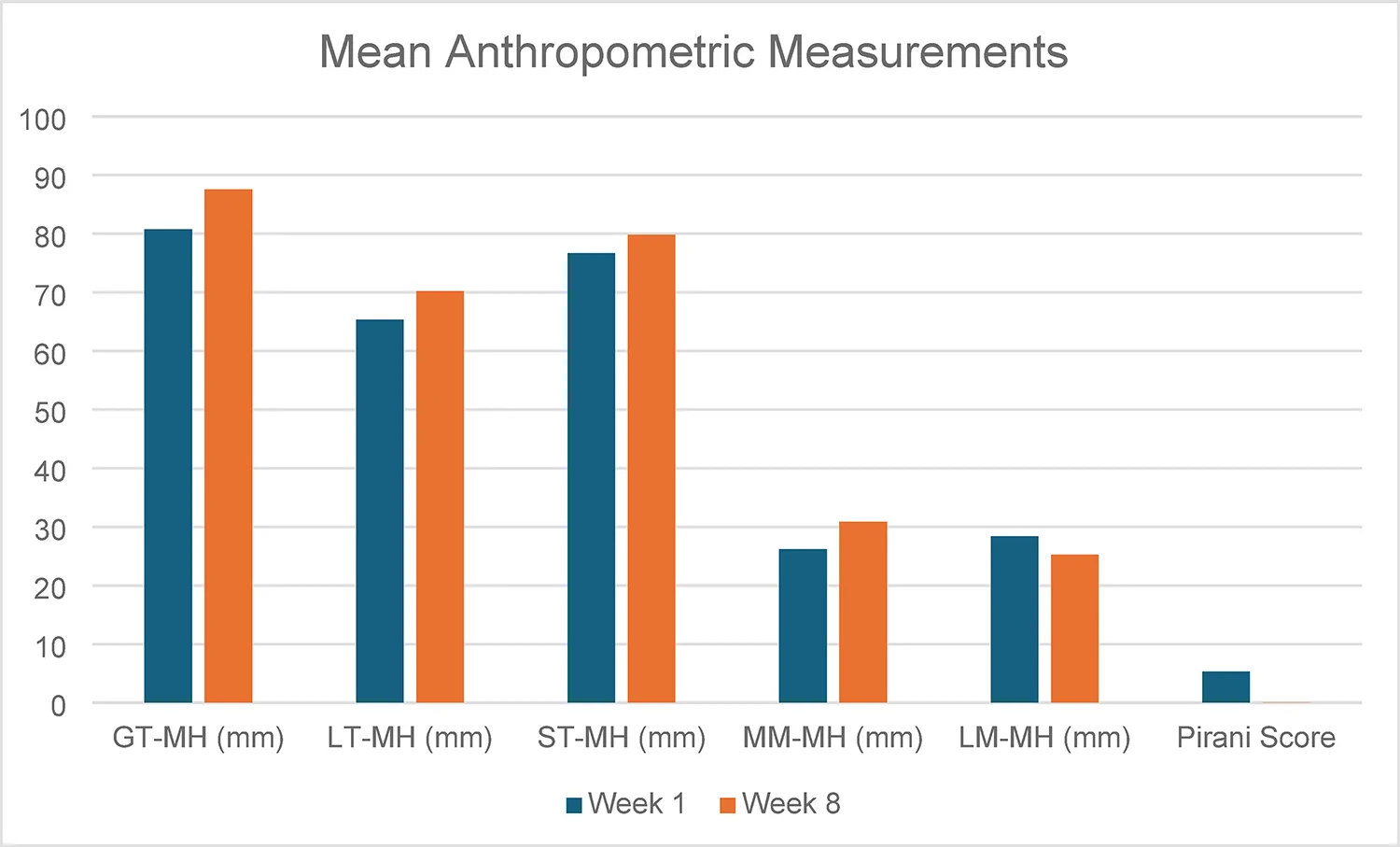
Comparison of Mean Anthropometric Measurements and Pirani Scores at Week 1 and Week 8

GT-MH increased from 80.73 ± 3.36 mm to 87.56 ± 3.41 mm (*p* < 0.001), while LT-MH increased from 65.36 ± 2.04 mm to 70.22 ± 2.05 mm (*p* < 0.001). MM-MH increased significantly from 26.19 ± 0.79 mm to 30.85 ± 0.78 mm (*p* < 0.001). In contrast, LM-MH decreased from 28.40 ± 0.56 mm to 25.25 ± 0.51 mm, indicating correction of hindfoot varus deformity (*p* < 0.001) (Table 1) (Fig. 3).

**Table 1:** Comparison of Anthropometric Measurements and Pirani Scores (Mean ± SD)

Parameter	Week 1	Week 8	Mean Diff	*p*-value
GT-MH (mm)	80.73 ± 3.36	87.56 ± 3.41	+ 6.83	< 0.001
LT-MH (mm)	65.36 ± 2.04	70.22 ± 2.05	+ 4.86	< 0.001
ST-MH (mm)	76.67 ± 1.02	79.83 ± 1.04	+ 3.16	< 0.001
MM-MH (mm)	26.19 ± 0.79	30.85 ± 0.78	+ 4.66	< 0.001
LM-MH (mm)	28.40 ± 0.56	25.25 ± 0.51	-3.14	< 0.001
Pirani Score	5.33 ± 0.24	0.03 ± 0.18	-5.3	< 0.001

Paired t-test; *p* < 0.05 significant

The mean Pirani score improved significantly from 5.33 ± 0.24 at baseline to 0.03 ± 0.18 at Week 8 (*p* < 0.001) (Table 1) (Fig. 3).

All anthropometric stature measurements show gradual weekly increase during Ponseti correction for GT-MH‚ LT-MH‚ ST-MH‚ MM-MH and gradual decrease in LM-MH indicating correction of hindfoot varus deformity․

**Fig. 4:**
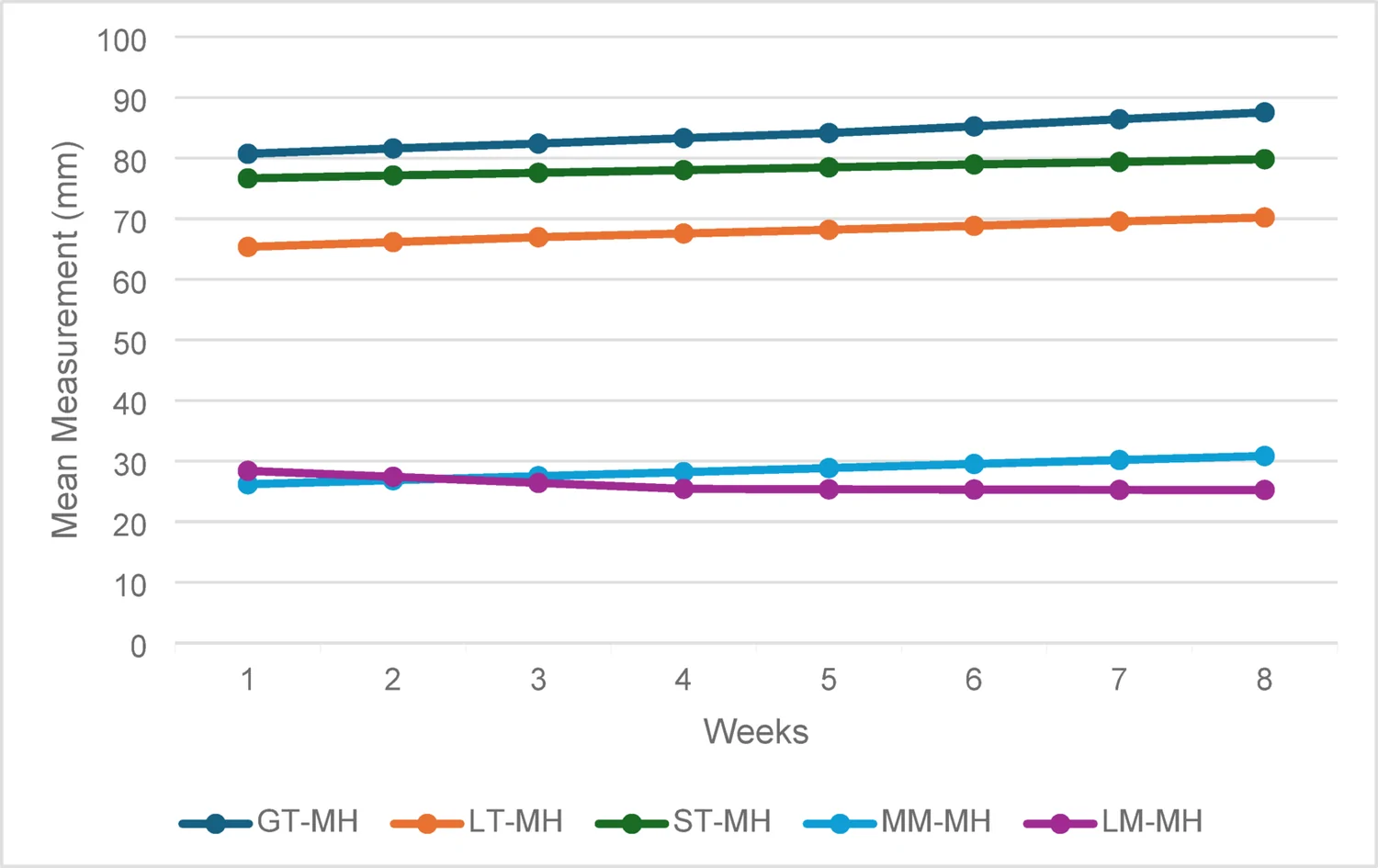
Trend of Mean Anthropometric Parameters Over 7 Weeks of Follow-Up

Maximum Pirani score change was seen in the first 4 weeks of treatment‚ at which point active deformity correction by serial casting is being achieved․ The average Pirani score decreased considerably from the mean value of 5․33 ± 0․24 at the beginning of treatment to 0․03 ± 0․18 at 8 weeks․ The maximum decrease in Pirani score was seen in the first 4 weeks (Table 2) (Fig. 4).

**Table 2:** Weekly Mean Progression of Anthropometric Parameters (mm)

Week	GT-MH	LT-MH	ST-MH	MM-MH	LM-MH	Pirani Score
1	80.73	65.36	76.67	26.19	28.40	5.33
2	81.61	66.15	77.15	26.86	27.42	4.83
3	82.41	66.96	77.58	27.52	26.41	4.30
4	83.31	67.57	78.03	28.19	25.43	3.65
5	84.15	68.18	78.48	28.86	25.35	2.72
6	85.25	68.83	78.97	29.54	25.32	1.70
7	86.43	69.56	79.38	30.19	25.26	0.85
8	87.56	70.22	79.83	30.85	25.25	0.03

Repeated measures ANOVA; *p* < 0.05 significant

**Fig. 5:**
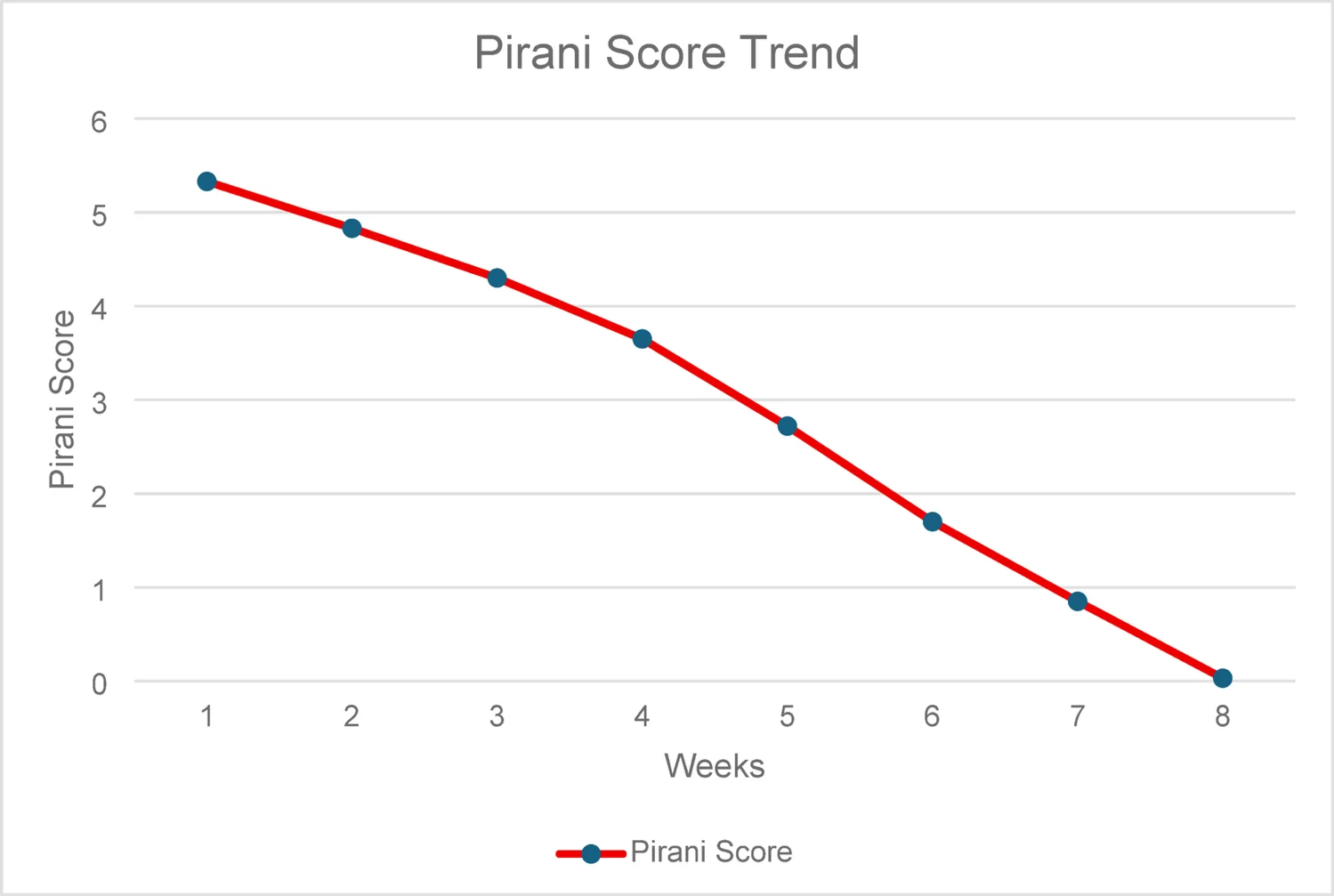
Trend of Mean Pirani Score Over 7 Weeks of Follow-Up

In general‚ weekly changes in serial anthropometric measures closely correlated with weekly changes in clinical improvement as measured by the Modified Pirani score (Table 2) (Fig. 5).

### Correlation analysis

GT-MH, LT-MH, ST-MH, and MM-MH demonstrated significant inverse correlations with Pirani scores, indicating improvement in anthropometric dimensions with decreasing deformity severity.

**Fig. 6:**
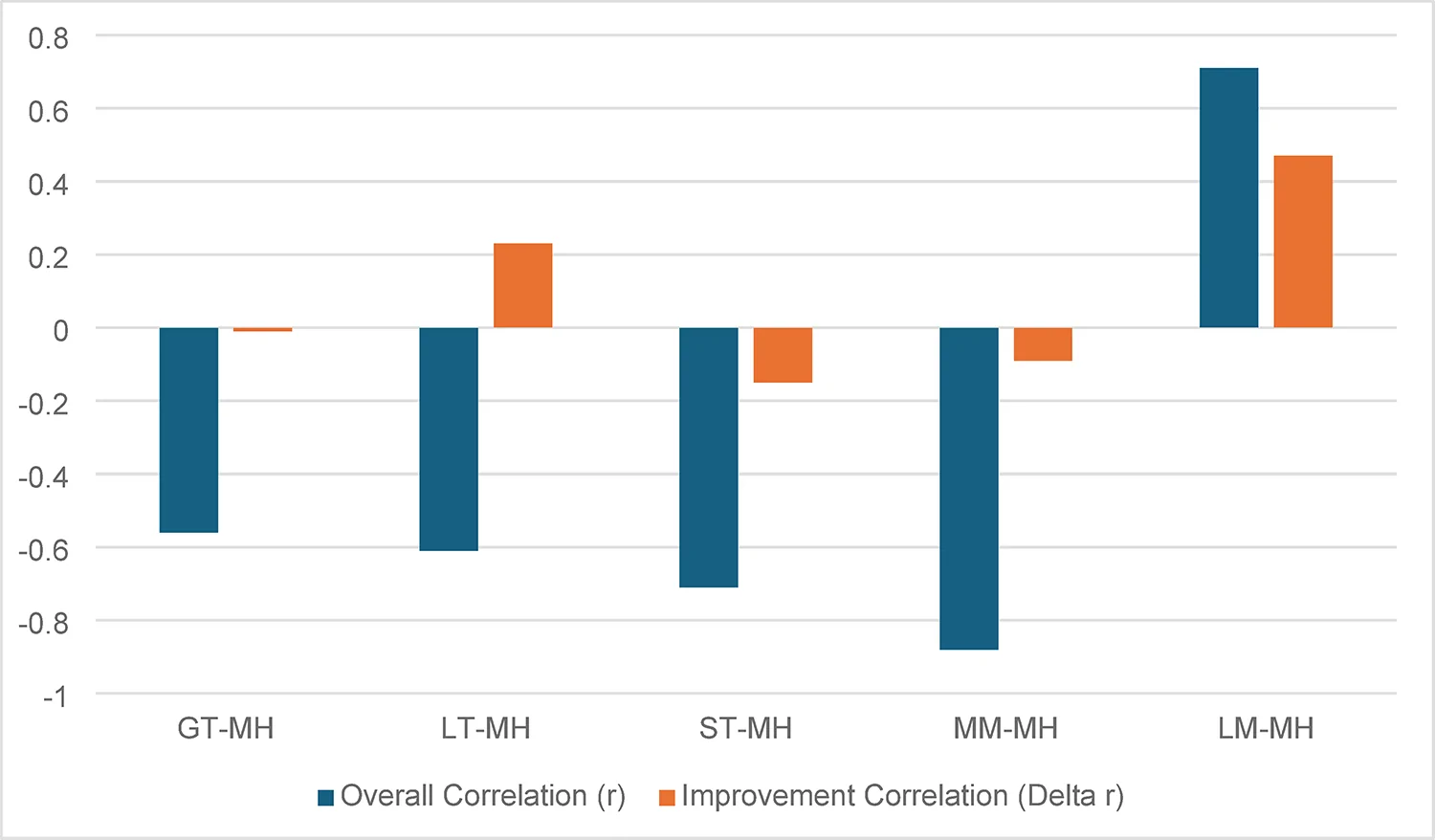
Pearson Correlation Coefficients Between Anthropometric Measurements and Pirani Score

The strongest inverse correlation was observed with MM-MH (r = -0.88; *p* < 0.001). LM-MH demonstrated a significant positive correlation with Pirani score (r = 0.71; *p* < 0.001), reflecting progressive reduction in lateral hindfoot displacement during correction (Table 3) (Fig. 6).

**Table 3:** Pearson Correlation Coefficients between Anthropometry and Pirani Score

Parameter	Overall Correlation (r)	*p*-value	Improvement Correlation (Delta r)	*p*-value
GT-MH	-0.56	< 0.001	-0.01	0.972
LT-MH	-0.61	< 0.001	0.23	0.229
ST-MH	-0.71	< 0.001	-0.15	0.426
MM-MH	-0.88	< 0.001	-0.09	0.634
LM-MH	0.71	< 0.001	0.47	0.008

Pearson correlation analysis; *p* < 0.05 significant

### Inter-observer reliability

Inter-observer reliability was excellent for all anthropometric measurements and Modified Pirani scoring, with intraclass correlation coefficients (ICC) ranging from 0.996 to 1.000, indicating excellent agreement between observers.

### Tenotomy requirement

Percutaneous Achilles tenotomy was required in 24 patients (80%), while 6 patients (20%) achieved correction without tenotomy.

### Analysis of intervention versus natural growth

In terms of anthropometric progressions‚ GT-MH increased by 6․83 mm‚ ST-MH increased by 3․16 mm‚ and LT-MH increased by 4․86 mm during the period of this study․ The larger increases in GT-MH and LT-MH compared to ST-MH suggest that the anthropometric changes during this time period were due to a treatment effect rather than physiological maturation (Table 4).

**Table 4:** Analysis of Medial Border Lengthening (Intervention vs. Growth)

Parameter	Mean Change (Week 1 to 8)	Contribution
ST-MH (Natural Growth)	3.16 mm	46.3%
GTMH-STMH (Intervention Effect)	3.67 mm	53.7%
GT-MH (Total Medial Gain)	6.83 mm	100%

### Correlation between clinical severity and correction index

**Fig. 7:**
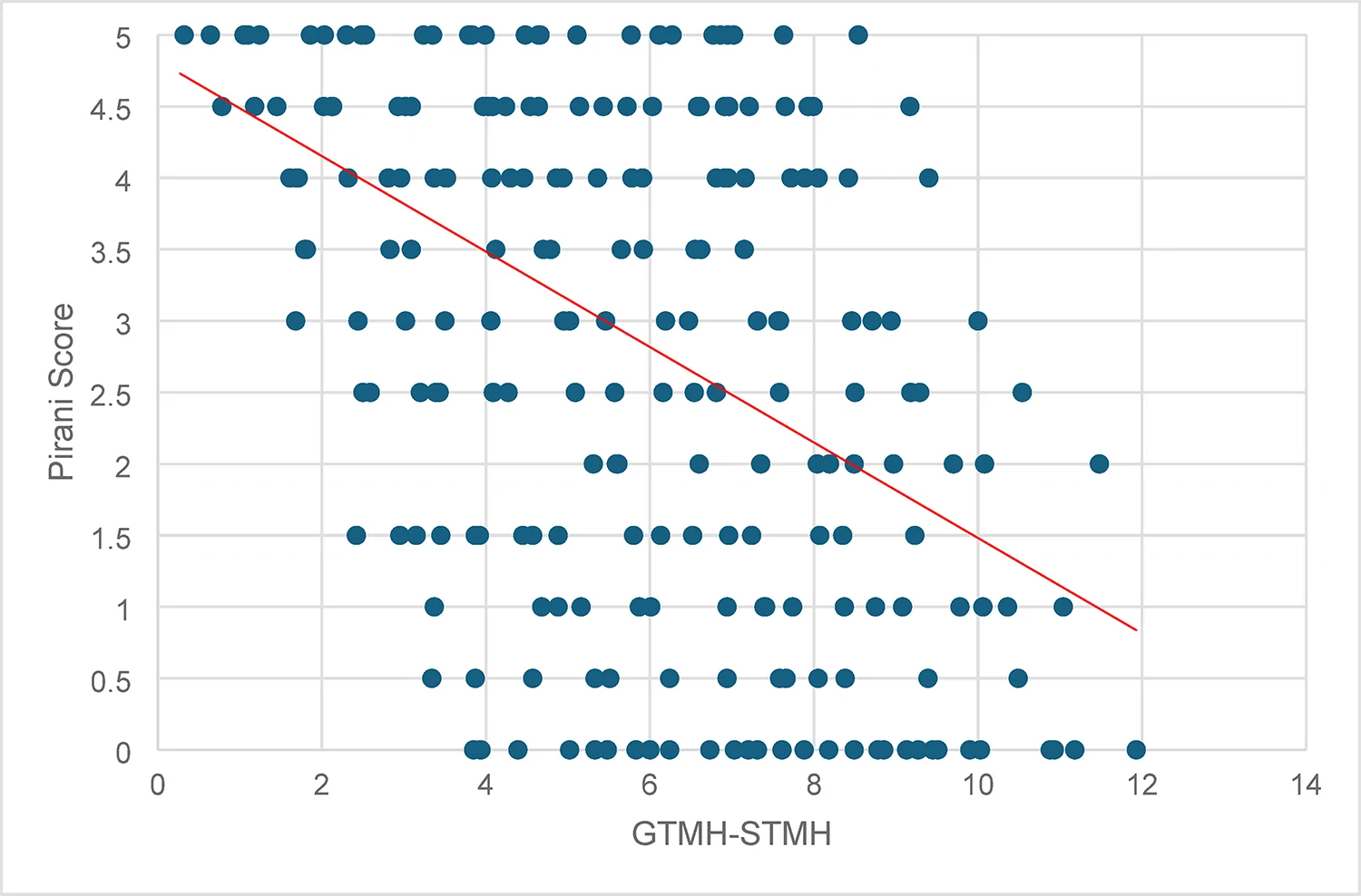
Scatter Plot- Correlation between GTM-STMH vs Pirani Score

Correlation analysis demonstrated a significant relationship between baseline clinical severity and correction index during treatment. Higher initial Pirani scores were associated with greater anthropometric change during serial correction. Scatter plot analysis demonstrated an inverse relationship between GT-MH/ST-MH ratio and Pirani score, indicating progressive improvement in foot anthropometry with reduction in deformity severity (Fig. 7).

## Discussion

In contrast to categorical clinical scoring systems, anthropometric measurements produce continuous quantitative data that can be sensitive enough to detect subtle changes during the treatment process [12]. The present study demonstrated significant improvement in both anthropometric foot measurements and Modified Pirani scores during Ponseti correction of idiopathic clubfoot. Over the eight-week treatment period, GT-MH increased from 80.73 ± 3.36 mm to 87.56 ± 3.41 mm, LT-MH from 65.36 ± 2.04 mm to 70.22 ± 2.05 mm, and MM-MH from 26.19 ± 0.79 mm to 30.85 ± 0.78 mm (p < 0.001 for all). In contrast, LM-MH decreased from 28.40 ± 0.56 mm to 25.25 ± 0.51 mm, reflecting progressive correction of hindfoot varus. Simultaneously, the mean Pirani score improved significantly from 5.33 ± 0.24 at baseline to 0.03 ± 0.18 at week 8 (*p* < 0.001). These findings indicate that serial anthropometric measurements closely parallel clinical correction during Ponseti treatment.

In clubfoot‚ anthropometric measures are based on the resultant structural changes․ In the untreated clubfoot‚ medial soft tissue contracture‚ forefoot adduction and hindfoot varus contribute to the shortening of the foot and aberrant alignment of the hindfoot․ Consistently abducting the forefoot around the head and neck of the talus and correcting hindfoot varus results in normal foot dimensions by the end of Ponseti manipulation․ GT-MH, LT-MH and MM-MH all considerably increased (foot length restored and adduction corrected)‚ and LM-MH considerably decreased (hindfoot corrected) with Ponseti manipulation․ The strong negative correlation (r =  − 0․88) between the MM-MH and Pirani score means that medial hindfoot reconversion is closely related to the reduction of deformity severity․ The strong positive correlation (r = 0․71) between LM-MH and Pirani score confirms the diagnostic value of the former in determining the degree of hindfoot varus correction․

It was also observed that the increase of GT-MH in the study period was larger than ST-MH․ The increase in ST-MH and GT-MH was 3․16 and 6․83 mm respectively‚ thus suggesting more than 50% of the increase of medial foot length was the result of the correction of deformity rather than physiological increase in length․ This evidence hence, supported that anthropometric measurements may objectively distinguish the result of treatment from the growth of normal infants․

These anthropometric changes can be explained by the Calcaneopedal Unit (CPU) concept, which forms the biomechanical basis of the Ponseti technique. The CPU comprises the calcaneus, navicular, cuboid, and forefoot, which function as a single unit rotating beneath the talus during manipulation. Progressive abduction and external rotation of the CPU around the talar head correct forefoot adduction, hindfoot varus, and cavus while preserving joint congruity.

The strong correlation between MM-MH and the Modified Pirani score is likely attributable to the close relationship between the medial malleolus and the hindfoot, which undergoes substantial realignment during Ponseti correction. As hindfoot varus is progressively corrected, the medial malleolus–midheel distance increases, closely reflecting restoration of hindfoot alignment. This finding suggests that MM-MH may be a particularly useful objective parameter for monitoring hindfoot correction during serial follow-up.

Consequently, serial increases in GT-MH, LT-MH, and MM-MH, together with a reduction in LM-MH, objectively reflect the progressive realignment of the CPU during deformity correction, providing a biomechanical explanation for the observed anthropometric changes.

These results are consistent with other studies that assess the relationship between the clinical severity and Ponseti outcome․ Ahmed HAD et al. [13] found that the initial Pirani score was the strongest predictor of the number of casts required to achieve correction (*p* < 0․001)‚ highlighting the importance of the severity of the deformity․ Similarly‚ Sharma et al. [14] noted 74․4% of the Ponseti corrected patients required Achilles tenotomy and found baseline severity scores to be predictive of tenotomy‚ as in our study․ In the present study, 80% of patients required tenotomy, which is consistent with these observations and reflects the persistence of hindfoot equinus despite correction of other deformity components.

In addition‚ the good inter-observer reproducibility (r > 0․99 for all parameters) in the current study shows that anthropometric measurements when performed in a standard manner are reliable and reproducible․ Since observer variability is a potential disadvantage of purely clinical scoring systems‚ anthropometric measurement might be a good adjunct to the Pirani scoring system in the follow-up of clubfoot deformity by improving the objectivity and reproducibility․

Anthropometric measurements provide objective, quantitative documentation of deformity correction and complement the Modified Pirani score by enabling reproducible monitoring during serial follow-up. Their simplicity and objectivity may be particularly useful for less experienced clinicians and for documenting treatment progression over time.

Barik and Agarwal [15] point out that some of the signs of Pirani‚ such as the "empty heel" sign may still be noted in clinical examination after correction․ They argue anthropometric improvement may reflect progressive structural correction despite apparently residual clinical signs․ This thus corroborates that anthropometric assessment cannot replace Pirani scoring‚ but should rather complement clinical evaluation of the treatment․

The greater increase observed in GT-MH compared with ST-MH (6.83 mm vs. 3.16 mm) suggests that deformity correction contributed substantially to the anthropometric changes observed during Ponseti treatment. Since ST-represents the adjacent forefoot ray and is relatively less affected by cavus and medial column shortening, it was used as an internal reference to estimate the contribution of physiological foot growth. However, this comparison should be interpreted cautiously, as ST-MH is a surrogate reference rather than a validated marker of physiological growth.

The clinical severity and the correction index were positively correlated: the feet with worse deformity on presentation had greater anthropometry changes during treatment․ This is supported by the correlation between the GT-MH/ST-MH ratio and the Pirani score‚ indicating that the foot shape change closely follows the clinical severity improvement of the deformity․ These findings suggest that anthropometric assessment may be useful to objectively assess structural correction during Ponseti treatment․

The strengths of the present study are its potential design‚ weekly serial assessment‚ objective measurement protocol‚ and assessment by a different observer․

However, the present study has certain limitations. The sample size was relatively small, and only children with unilateral idiopathic clubfoot were included, limiting generalizability. Follow-up was restricted to the casting phase; therefore, long-term recurrence and functional outcomes were not assessed. Although Pearson correlation was used to evaluate the association between anthropometric measurements and Modified Pirani scores, it does not account for the within-subject correlation inherent in repeated measurements. Future studies using longitudinal statistical models may provide more robust estimates. Additionally, as the study included children younger than two years of age, age-related physiological foot growth may have influenced the observed anthropometric changes despite serial assessments. Finally, the absence of measurements from the contralateral normal foot or healthy controls precluded direct comparison with normal foot growth and anthropometric development during the study period.

## Conclusions

Foot anthropometric measurements provide objective quantitative information that complements the Modified Pirani scoring system during Ponseti treatment. They may serve as a useful adjunct for monitoring deformity correction and documenting treatment progression during serial follow-up.

## Data Availability

The datasets used and/or analyzed during the current study are available from the corresponding author on reasonable request.

## Abbreviations

CTEV: Congenital talipes equinovarus
GT-MH: Great toe to midheel distance
LT-MH: Little toe to midheel distance
ST-MH: Second toe to midheel distance
MM-MH: Medial malleolus to midheel distance
LM-MH: Lateral malleolus to midheel distance
ICC: Intra-class correlation coefficient

## References

[1] Barnes CJ, Dydyk AM (2026) Talipes equinovarus. In: StatPearls [Internet]. StatPearls Publishing, Treasure Island 32491773

[2] Smythe T, Kuper H, Macleod D, Foster A, Lavy C (2017) Birth prevalence of congenital talipes equinovarus in low- and middle-income countries: a systematic review and meta-analysis. Trop Med Int Health 22(3):269–285 28000394 10.1111/tmi.12833

[3] Alves C, Batlle AE, Rodriguez MV (2021) Neglected clubfoot treated by serial casting: a narrative review on how possibility takes over disability. Ann Transl Med 9(13):1103 34423015 10.21037/atm-21-65 PMC8339819

[4] Dobbs MB, Nunley R, Schoenecker PL (2006) Long-term follow-up of patients with clubfeet treated with extensive soft-tissue release. J Bone Joint Surg Am 88(5):986–996 16651573 10.2106/JBJS.E.00114

[5] Radler C (2013) The Ponseti method for the treatment of congenital club foot: review of the current literature and treatment recommendations. Int Orthop 37(9):1747–1753 23928728 10.1007/s00264-013-2031-1 PMC3764299

[6] Morcuende JA, Dolan LA, Dietz FR, Ponseti IV (2004) Radical reduction in the rate of extensive corrective surgery for clubfoot using the Ponseti method. Pediatrics 113(2):376–380 14754952 10.1542/peds.113.2.376

[7] Mosca VS (2021) Clubfoot pathoanatomy-biomechanics of deformity correction: a narrative review. Ann Transl Med 9(13):1096. 10.21037/atm-20-7491 34423008 10.21037/atm-20-7491 PMC8339823

[8] Dyer PJ, Davis N (2006) The role of the Pirani scoring system in the management of club foot by the Ponseti method. J Bone Joint Surg Br 88(8):1082–1084 16877610 10.1302/0301-620X.88B8.17482

[9] Radler C, Manner HM, Suda R, Burghardt R, Herzenberg JE, Ganger R, Grill F (2007) Radiographic evaluation of idiopathic clubfeet undergoing Ponseti treatment. J Bone Joint Surg Am 89(6):1177–1183. 10.2106/JBJS.F.00438 17545419 10.2106/JBJS.F.00438

[10] Agarwal A, Rastogi A (2018) Anthropometric measurements in Ponseti treated clubfeet. SICOT J 4:19. 10.1051/sicotj/2018010 29806584 10.1051/sicotj/2018010 PMC5971664

[11] Jain AK, Zulfiqar AM, Kumar S, Dhammi IK (2001) Evaluation of foot bimalleolar angle in the management of congenital talipes equinovarus. J Pediatr Orthop 21(1):55–59. 10.1097/00004694-200101000-00012 11176354 10.1097/00004694-200101000-00012

[12] Kesemenli CC, Kapukaya A, Subaşi M, Necmioglu S, Arslan H, Ozbag D, Celik Y (2003) Anthropometric study of patients treated for clubfoot. J Pediatr Orthop 23(4):498–502 (**PMID: 12826950**) 12826950

[13] Ahmed HAD, Mohamed AMY, Salih M, Mohamed MMG, Younis A, Hussein SHM, Sovla H, SeedAhmed LMK (2024) Assessing the influence of age, weight, and Pirani score on the number of casts during the initial phase of clubfoot treatment using the Ponseti method: a prospective study. Indian J Orthop 58(6):687–695. 10.1007/s43465-024-01142-2 38812862 10.1007/s43465-024-01142-2 PMC11130105

[14] Sharma S, Banskota B, Yadav P, Rajbhandari T, Bhusal R, Banskota AK (2023) Factors predictive of tenotomy after Ponseti casting for idiopathic clubfoot: a tertiary care center study. J Pediatr Orthop 43(3):174–176. 10.1097/BPO.0000000000002338 36728662 10.1097/BPO.0000000000002338

[15] Barik S, Agarwal A (2023) Non-zero Pirani score in corrected clubfoot due to empty heel: a prognostic dilemma. J Clin Orthop Trauma 2(47):102295. 10.1016/j.jcot.2023.102295 10.1016/j.jcot.2023.102295 PMC10772365 38196502

